# Bacterial urinary tract infection among adult renal transplant recipients at St. Paul’s hospital millennium medical college, Addis Ababa, Ethiopia

**DOI:** 10.1186/s12882-019-1485-9

**Published:** 2019-07-31

**Authors:** Teklehaimanot Kiros, Daniel Asrat, Zeleke Ayenew, Estifanos Tsige

**Affiliations:** 1 Department of Medical Microbiology, College of Medicine and Health Sciences, Debre Tabor University, Debre Tabor, Ethiopia; 2 Department of Microbiology, Immunology and Parasitology, School of Medicine, College of Health Sciences, Addis Ababa University, Addis Ababa, Ethiopia; 3 Ethiopian Public Health Institute, Addis Ababa, Ethiopia

**Keywords:** Kidney transplantation, Urinary tract infection, Urine culture, Antimicrobial susceptibility testing, Addis Ababa, Ethiopia

## Abstract

**Background:**

Despite significant advances in surgical techniques, immunosuppression protocols, follow up periods and antimicrobial stewardship in modern medicine; post-renal transplantation urinary tract infection remained a major public health problem globally. This multiple serious squeals includes asymptomatic bacteriuria, cystitis and pyelonephritis. Among these, the bacterial origin of infection complications accounts for the most significant clinical, socio-economic impacts in many countries of the world. Therefore, the aim of the study was to investigate the prevalence of bacterial isolates that cause urinary tract infections, assess antibiotic susceptibility pattern among symptomatic and asymptomatic renal transplant recipients attending at St. Paul’s Hospital Millennium Medical College, Addis Ababa, Ethiopia.

**Methods:**

A hospital-based cross-sectional study was conducted from December 2017 to August 2018 among 74 renal transplant recipients St. Paul’s Hospital Millennium Medical College, Addis Ababa, Ethiopia. A first morning voided clean-catch mid-stream urine specimens were collected and 0.001 ml inoculated onto blood and MacConkey agar plates following the standard bacteriological protocols. It was incubated aerobically at 35–37 °C for 24–48 h. Cultural characteristics and series of biochemical tests were used for the identification of isolates to species level based on the standard bacteriological protocols.

**Results:**

A hospital-based cross-sectional study has shown that significant bacteriuria was found in 11/74 (14.9, 95% CI =8.2–24.7) patients. The prevalence among females 6/32 (18.75%) was higher among males 5/42 (11.9%) without significant association (COR = 2.09, 95% CI = 1.04–8.45, *P* = 0.253). Urinary tract infection was higher in the age group of 35–49 years old (19.3%). Age was statistically significant and stronger independent associated risk factor with crude odds ratio = 3.67, 95% CI = 2.89–20.07 and *P* = 0.003, respectively. The most prevalent bacteria isolates were *Escherichia coli* 2(18.2%), *Staphylococcus aureus* 2(18.2%)*, Acinetobacter spp*. 2(18.2%), *Enterococcus spp*. 2(18.2%), Coagulase-negative *Staphylococci 2*(18.2%) followed by *Porteus mirabilis* 1(9.1%).

The majority (80%) of Gram-negative bacteria were resistant to ciprofloxacin, chloramphenicol, and trimethoprim/sulfamethoxazole. Simultaneously, the multidrug-resistant bacterial isolates accounts for 82% among tested kidney allograft recipients.

**Conclusions:**

In conclusion, the overall prevalence of urinary tract infection in the study participants was relatively low with a prevalence of 14.9%. Majority of the study participants were asymptomatic and a higher percentage of females were involved. The multidrug-resistant bacterial isolates in the present study account for 82%.

**Electronic supplementary material:**

The online version of this article (10.1186/s12882-019-1485-9) contains supplementary material, which is available to authorized users.

## Background

Urinary tract is protected against infections by several mechanisms unlike the kidney transplant patients are not. Despite significant advances in surgical techniques and immunosuppression, post-renal transplantation urinary tract infections particularly the bacterial origin continue to be a major public health problem globally with significant morbidity and mortality [1]. Post-renal transplantation urinary tract infections squeal including asymptomatic bacteriuria, cystitis, and pyelonephritis are the most common form of bacterial infection following renal transplantation. These can occur at any time but with the highest incidence in the first 3–6 months after transplantation [2].

The globally reported prevalence of post- renal transplantation urinary tract infection may vary depending the study design, immune suppression protocol, surgical protocol and diversity in the use of antimicrobial prophylaxis. However, incidence varies depending on the type of transplanted organ being the renal transplants are the highest risk groups among any other organ transplants [3–5].

In many countries of the world, kidneys are the most frequently transplanted organs to resolve end-stage renal disease. However, post-renal transplantation urinary tract infection has remained the leading cause of significant morbidity, mortality and graft failure, which reported globally. The clinical and socio-economic impacts are relatively higher in developing countries [6, 7]. The super imposed immune suppressions aimed to maintain the acute or chronic allograft rejection triggers antimicrobial selective pressure. In general, bacterial species leading to urinary tract infection in renal transplant recipients are similar to those causing UTIs in the general population. However, management in renal transplant recipients is undoubtedly more complex compared with the general population [8, 9]. The frequency of UTIs depends on many factors such as age, female gender, co-morbidities, immunosuppressive protocol and follow-up period [3, 10–14].

## Methods

### Study design, area and period

A hospital-based cross-sectional study was conducted between December 2017 and August 2018 at St. Paul’s Hospital Millennium Medical College at the National Kidney Transplantation Center.

### Study population

The study population were all adult kidney recipients aged ≥18 years who came for their check-up to the renal transplantation center suspected for both asymptomatic, symptomatic bacteriuria and who did not initiate of antibiotics therapy during the last 2 weeks and during data collection.

### Sample collection

Seventy-four early morning 5 ml of midstream urine specimens were collected from all kidney recipients using wide-mouthed, sterile, leak-proof re-usable plastic containers following standard bacteriological procedures. All relevant data concerning socio-demographic characteristics, related risk factors to UTI, clinical signs and symptoms of the study participants were obtained using pre-designed structured questionnaires.

### Bacterial culture and identifications

Relevant data on the etiological agents were obtained using standard microbiological laboratory tests. The laboratory procedures were performed at clinical Bacteriology and Mycology laboratory located at National Reference Laboratory of the Ethiopian Public Health Institute, Addis Ababa, Ethiopia. All the laboratory procedures were performed using standard bacteriological procedures. Briefly, using calibrated wire loop One μl (0.001 ml) clean-catch midstream urine samples were inoculated into MacConkey (MAC) and 5% sheep blood agar plate (BAP) (Oxoid, UK). Then, cultures were incubated in the aerobic atmosphere at 35–37 °C for 24–48 h. Colonies were counted to check the presence of significant bacteriuria. Colony count yielding bacterial growth of ≥10^5^ cfu/ml of urine was considered significant bacteriuria according to the Infectious Diseases Society of America (IDSA) guidelines [15]. All positive cultures with significant bacteriuria were then subjected to test identification to species level by their colony characteristics and patterns of biochemical profiles using standard bacteriological procedures [3, 16].

### Antimicrobial susceptibility testing

Antibiotic susceptibility testing was performed for every significant positive culture following the manufacturer’s instructions. Mueller-Hinton agar (Oxoid, UK) was used to do the susceptibility testing for the isolated bacteria. Isolates were classified as sensitive, intermediate and resistant according to the criteria of CLSI [17].

### Data quality control

The quality of culture media was tested for sterility and performance. Sterility of culture media was checked by incubating overnight at 35–37 °C without specimen inoculation. Standard reference strains of *E. coli* (ATCC 25922), *S. aureus* (ATCC 25923) and *P. aeruginosa* (ATCC 27853) were used for quality control throughout the study for culture and antimicrobial susceptibility test.

### Data management and statistical analysis

All the patient’s records were anonymized by giving a number to each sample and questionnaire before the analysis and secured at all levels. All data were analyzed taking due to care for completeness, consistency, coding and sorting using SPSS (Statistical Package for Social Sciences) computer program (Version 20.0). Then, tables and texts were utilized to explain the descriptive data. In all cases, *P*-value < 0.05 was taken as statistically significant. Furthermore, to assess any associated risk factors for post-renal transplant UTI, bivariate and multivariate logistic regression risk factor analysis was done to calculate crude/adjusted odds ratio and 95% confidence interval.

### Ethical considerations

Ethical approval was obtained from the Department Ethics Research Committee (DERC), Department of Microbiology, Immunology, and Parasitology, School of Medicine, College of Health Sciences, Addis Ababa University (DERC committee’s reference number: DERC/17/18/02-C). Subsequently, ethical approval was also obtained from St. Paul’s Hospital Millennium Medical College (SPHMMC) Institutional Review Board (IRB reference number: P.m 23/409). Finally, the study secures at all levels and study participants were informed about the objective and benefit preceding the data collection procedure.

## Results

### Socio-demographic characteristics of studied participants

A total of 74 study participants (38 with symptoms and signs of UTI and 36 without symptoms and signs of UTI) were included in the study at St. Paul’s Hospital Millennium Medical College. A majority, 42/74(56.8%) of them were males. The mean age was 41.55 years old with a standard deviation of 11.33 (41.55 ± 11.33) and a median of 40.5. Majority of the study participants 31(41.9%) were within the age group of 35–49 followed by 18–34(29/74, 39.2%) (Table 1).

**Table 1:** Sociodemographic characteristics of study participants with and without UTI, St Paul’s Hospital Millennium medical college, Addis Ababa, Ethiopia

Variables	Total (%)	UTI no (%)	No UTI no (%)	Bivariate analysis		*P*-value
				COR	95%CI	
Gender						
Male	42 (56.8)	5 (11.9)	37 (88.1)	0.848	0.57–11.31	0.419
Female	32 (43.2)	6 (18.75)	26 (81)	2.09	1.04–8.45	0.253
Age						
18–34	29 (39.2)	4 (13.8)	25 (86.2)	1.42	0.64–14.05	0.338
35–49	31 (41.9)	6 (19.3)	25 (80.6)	3.67	2.89–20.07	0.003
50–64	10 (13.5)	1 (10)	9 (90)	3	2.91–10.00	0.914
Above 64	4 (5.4)	0 (0.0)	4 (100)	0.88	0.58–3.27	0.444
Marital status						
Single	23 (31)	6 (26.1)	17 (74)	5.64	0.73–13.22	0.222
Married	30 (40.5)	4 (13.3)	26 (86.7)	1.724	1.081–6.82	0.391
Divorced	12 (16.2)	1 (8.3)	11 (91.7)	7	4.36–9.15	0.284
Widowed	5 (6.8)	0 (0.0)	5 (100)	0.23	0.11–3.74	0.058
Widower	4 (5.4)	0 (0.0)	4 (100)	8.04	2.05–10.09	0.701
Educational level						
Student	11 (14.9)	2 (18.2)	10 (91)	1	0.37–4.12	0.348
Diploma	31 (41)	5 (16)	26 (83.9)	2.872	0.81–5.06	0.579
Degree	13 (17.6)	2 (15)	11 (84.6)	4	2.01–6.19	0.441
Illiterate	15 (20)	2 (13)	13 (86.7)	2.11	1.90–17.48	0.990
Above degree	4 (5.4)	0 (0.0)	4 (100)	0.81	0.36–1.34	0.007

### Clinical characteristics of study participants

The average time since transplantation in months was 38.4 ± 4.8 (Table 2).

**Table 2:** Prevalence of UTI in related clinical variables of renal transplants recipients

Variables	Total (%)	UTI no (%)	No UTI no (%)	Bivariate analysis		*p*-value
				COR	95%CI	
Time since transplantation						
0–6 months	17 (22.9)	3 (17.6)	14 (82.3)	2.29	0.42–2.96	0.391
7–12 months	19 (25.7)	4 (21)	15 (79)	2.57	1.09–11.03	0.555
13–24 months	19 (25.7)	2 (10.5)	17 (89.5)	0.71	0.54–6.38	0.081
> 24 months	19 (25.7)	2 (10.5)	17 (98.5)	1.23	0.98–7/11	0.661
Pre- transplant UTI history						
Yes	5 (6.8)	2 (40)	3 (60)	4.32	2.09–17.10	0.010
No	69 (93.2)	9 (13)	60 (87)	0.51	0.26–2.11	0.997
Place of the transplantation						
Local	54 (73)	9 (16.7)	45 (83.3)	4.01	0.18–19.06	0.481
Abroad	20 (27)	2 (10)	18 (90)	0.89	0.69–8.81	0.671
Donor’s gender						
Male	40 (54.1)	5 (12.5)	35 (87.5)	3.1	2.19–3.70	0.561
Female	34 (45.9)	4 (11.8)	30 (88.2)	2.07	1.04–7.31	0.549
History of Catheterization						
Yes	5 (6.8)	1 (20)	4 (80)	1.90	1.11–11.38	0.001
No	69 (93.2)	10 (14.5)	59 (85.5)	0.53	0.21–0.98	0.941

### Prevalence of significant bacteriuria among renal transplant recipients

In the present study, significant bacteriuria was detected in 11/74 (14.9%) of the study participants investigated for urinary tract infection. In the meantime, the magnitude of significant bacteriuria has shown no association with the clinical signs and symptoms for post-renal transplantation urinary tract infection (Additional file 1: Table S1). *E.coli*, *P.mirabilis* and *Acinetobacter spp*. were exclusively found in asymptomatic patients (Additional file 2: Table S2).

### Bacterial etiologies

A total of 11 bacteria (Table 3) were isolated, out of these, 5 (45.4%) were Gram-negative bacteria and 6 (54.6%) were Gram-positive bacteria.

**Table 3:** Bacterial etiologic agents isolated from urine culture of renal transplants

Bacterial isolates	Frequency(n)	Percent (%)
Gram-Negative	5	45.4
*E.coli*	2	18.2
*Acinetobacter spp.*	2	18.2
*P.mirabilis*	1	9.1
Gram-positive	6	54.6
*Enterococcus spp.*	2	18.2
CoNS	2	18.2
*S.aureus*	2	18.2
Total	11	100

*CoNS* Coagulase-Negative *Staphylococci*

### Antibiotic susceptibility data

Clindamycin (67%) as shown in (Additional file 3: Table S3) and Gentamicin (100%) as shown in (Additional file 4: Table S4) were the most effective antibiotic among the groups against the Gram-positive and negative bacterial isolates respectively. Multidrug resistance (resistance in ≥3 drugs) was seen in 82% of the isolates among diagnosed renal transplant recipients.

## Discussion

Urinary tract infections mainly the bacterial origin are the most common infectious complication especially to kidney transplant recipients [18, 19]. Both formidable and none formidable associated risk factors are the leading consequences to either the allograft survival or patient survival besides the socioeconomic burdens. Influence of immunosuppression that leave the patient immune quell are top priority in many clinical settings [20–22].

The present study revealed that the majority (56.8%) of study participants were males. However, higher number of females were affected by post -renal transplantation UTI than males (18.75% versus 11.9% respectively) with insignificant association. In harmony to the present study, a research paper by Kotagiri et al., [23] in Australia, Shams et al.*,* [24] in Iran and Bispo et al.*,* [25] in Portugal has shown that a large number of females were affected (*P* = 0.002, *P* < 0.001 and *P* < 0.005 respectively). Unlike to the present finding, study from Yemen by Gondos et al., [14], Portugal by Bispo et al.*,* [25] and Saudi Arabia by Alkatheri, [26] higher female prevalence of UTI (female 40.3%, males 29%, female 68%, male 23% and female 69.2%, male 30.8% respectively) were reported with no statistically significant association. This may be due to women are more susceptible to UTIs, which results from anatomical, hormonal, immunological and behavioral features [27–30].

In the present study, the overall bacterial UTI was found 14.9% of the patients (95% CI = 8.2–24.7). The present prevalence was quite smaller than the recent reports from different parts of the world. Shams et al.*,* [24] in Iran, Becerra et al., [29] in the USA, Menegueti et al., [31] in Brazil, Elkehili et al., [32] in Libya and Ooms et al., [33] in Netherland reported as 22.7, 28, 26.2, 29.5 and 28%, respectively. On the other way, the highest incidence of UTI among renal transplant recipients was also reported by Khosravi et al., [7] in Iran, Gondos et al., [14] in Yemen, Alkatheri, [26] in Saudi Arabia that was 33.56, 33.5 and 55.5% respectively. However, the current result was nearly similar to reported results from Portugal (16.5%) by Bispo et al., [25] but much higher than the report by Kotagiri et al., [23] in Australia (8%). This significant variation in UTI reported rates might be due to local ascribe of outbreaks, center-specific potent immunosuppressive therapy, lack of the robust definition of UTI and study designs in many clinical settings [34].

In the present study, the multivariate logistic regression has shown that 35–49 age groups (*P* = < 0.001, adjusted odds ratio = 2.61, 95%CI = 2.06–18.19), the previous history of pre-transplantation UTI (*P* = 0.02, adjusted odds ratio = 3.48, 95%CI = 2.12–9.38) and the previous history of catheterization (*P* = 0.003, adjusted odds ratio = 3.29, 95% CI = 2.05–11.85) were associated risk factor. In line to the present report Bispo et al., [25] and Kumar et al.*,* [30] have shown the presence of pre-transplant UTI history as a risk factor for post-transplant UTI. Discordant to the present finding, Ooms et al., [33] unveiled that older age groups (> 65 years old) were the risk factors for post renal transplantation UTI (*P* = < 0.001, AOR = 3.58, 95%CI = 2.16–5.91). This discrepancy may be due to study design and impact of potent immunosuppressive drugs [35, 36].

In the contemporary study, the most prevalent bacteria isolates causing post-renal transplant UTI were *Escherichia coli* (18.18%), *Acinetobacter spp*.(18.18%), *P. mirabilis* (9.1%), *S. aureus* (18.18%,), *Enterococcus spp*. (18.18%), Coagulase-negative *Staphylococci* (18.18%). This result is incomparable with recently published research paper by Gozdowska et al., [1]); *E.coli* (42%) and *Enterococcus spp*. (10%). Similarly, the current finding dissimilar to a retrospective study done by Kotagiri et al., [23] that found *E.coli* (32%) and *Enterococcus spp*.(35%) which were responsible for post- renal transplantation UTI. In addition, another study unveils that *E. coli* (46%), *P. mirabilis* (26%), *S. aureus* (25.8%) and Coagulase-negative *Staphylococci* (6.8%) were etiologies of post-renal transplantation UTI which were relatively higher than the present result except to Coagulase-negative *Staphylococci* [37, 38]. The present finding was discordant with Elkehili et al., [32] that ciprofloxacin (51.6%), followed by amoxicillin-clavulanic acid (22.6) were choices of drug for the Gram-negative. This could be justified by bacterial antibiotic prophylaxis selection should have adhered to conventional urinary culture so that prophylaxis should be tailored based on appropriate antibiogram batteries. In addition, disparities with present findings may be due to the lack of access of antibiotics, selection of antimicrobial agents and antibiotic stewardship program [14, 39–43].

In the present study, multi drug-resistant strains was seen in 82% of the isolated bacteria. This is similar to the current study done by Yuan et al., [39] in China, which reported 86.4%. Gozdowska et al., [1] and Bodro et al., [41] were reported much lower than our finding (37%). This is a threat to kidney transplants because it increases health care costs, prolongs hospital stays and can result in treatment failure [44–46].

## Conclusions

In conclusion, the overall prevalence of UTI in our population was relatively low with a prevalence of 14.9%. Majority of the UTIs were asymptomatic. A higher percentage of females were involved. Intensive longitudinal research activities to identify the risk factors as well as to elucidate the existing controversies of post-renal-transplantation UTI over allograft outcome are highly demanding. In countries like Ethiopia where the resources are limited especially of advanced diagnostic facilities to screen and monitor renal transplants, it is better to establish routine urine cultures especially in the first 6–12 months after kidney transplantation for recipients on follow up. The current study is indicating the evolution of multidrug-resistant isolates among kidney transplants. To endorse judicious treatment, careful and systemic selection of antimicrobial agents together with rigorous infection preventions and control strategies should be employed to mitigate both hospital and community-acquired Urinary tract infections.

## Additional files

Additional file 1: **Table S1.** Significant bacteriuria from urine culture of renal transplant recipients. (DOCX 14 kb)

Additional file 2: **Table S2.** Bacterial species isolated from asymptomatic and symptomatic UTI among renal transplant recipients, St Paul’s Hospital Millennium medical college, Addis Ababa, Ethiopia. (DOCX 14 kb)

Additional file 3: **Table S3.** Antibacterial susceptibility patterns of Gram-positive bacterial isolates. (DOCX 17 kb)

Additional file 4: **Table S4.** Antibacterial susceptibility patterns of Gram-negative bacteria isolates. (DOCX 17 kb)

## Data Availability

All the available data and material used in this study is presented in the main paper.

## Abbreviations

ATCC: American Type Culture Collection
CLSI: Clinical and Laboratory Standard Institute
IDSA: Infectious Diseases Society of America
SPHMMC: St. Paul’s Hospital Millennium Medical College
SPSS: Statistical Package for Social Science
UTI: Urinary Tract Infection

## References

[1] Gozdowska J, Czerwińska M, Młynarczyk G, Kwiatkowski A, Chmura A, Durlik M (2016). Urinary tract infections in kidney transplant recipients hospitalized at a transplantation and nephrology ward: 1-year follow-up. Transplant Proc.

[2] Parasuraman R, Julian K (2013). Urinary tract infections in solid organ transplantation. Am J Transplant.

[3] Fiorentino M, Pesce F, Schena A, Simone S, Castellano G, Gesualdo L. Updates on urinary tract infections in kidney transplantation. J Nephrol. 2019:1–11. 10.1007/s40620-019-00585-3. 10.1007/s40620-019-00585-3 30689126

[4] Fontser S, Chacón N (2017). And Cordero. Review of bacterial urinary tract infection in kidney transplant recipients: incidence, risk factors and impact on the graft survival. Int J Transplant Res Med.

[5] Vidal E, Torre-Cisneros J, Blanes M, Montejo M, Cervera C, Aguado JM (2012). Bacterial urinary tract infection after solid organ transplantation in the RESITRA cohort. Transpl Infect Dis.

[6] Origüen J, López-Medrano F, Fernández-Ruiz M, Polanco N, Gutiérrez E, González E (2016). Should asymptomatic bacteriuria be systematically treated in kidney transplant recipients? Results from a randomized controlled trial. Am J Transplant.

[7] Khosravi AD, Montazeri EA, Ghorbani A, Parhizgari N (2014). Bacterial urinary tract infection in renal transplant recipients and their antibiotic resistance pattern: a four-year study. I J Microbiol.

[8] Säemann M, Hörl WH (2008). Urinary tract infection in renal transplant recipients. Eur J Clin Investig.

[9] Goldman JD, Julian K, AST Infectious Diseases Community of Practice. Urinary tract infections in solid organ transplant recipients: Guidelines from the American Society of Transplantation Infectious Diseases Community of Practice. Clin Transplant. 2019:e13507. 10.1111/ctr.13507. 10.1111/ctr.13507 30793386

[10] Hollyer I, Ison MG (2018). The challenge of urinary tract infections in renal transplant recipients. Transpl Infect Dis.

[11] Chuang P, Parikh CR, Langone A (2005). Urinary tract infections after renal transplantation: a retrospective review at two US transplant centers. Clin Transpl.

[12] Ariza-Heredia EJ, Beam E, Lesnick TG, Cosio F, Kremers WK, Razonable R (2014). Impact of urinary tract infection on allograft function after kidney transplantation. Clin Transpl.

[13] Coussement J, Abramowicz D (2014). Should we treat asymptomatic bacteriuria after renal transplantation?. Nephrol Dial Transplant.

[14] Gondos AS, Al-Moyed KA, Al-Robasi AB, Al-Shamahy HA, Alyousefi NA (2015). Urinary tract infection among renal transplant recipients in Yemen. PLoS One.

[15] Nicolle Lindsay E., Bradley Suzanne, Colgan Richard, Rice James C., Schaeffer Anthony, Hooton Thomas M. (2005). Infectious Diseases Society of America Guidelines for the Diagnosis and Treatment of Asymptomatic Bacteriuria in Adults. Clinical Infectious Diseases.

[16] Leber M. Clinical microbiology procedures hand book. 4tth ed: ASM; 2018, 2018.

[17] CLSI (2018). Performance standards for antimicrobial susceptibility testing. 28th ed. CLSI supplement M100.

[18] Shahid Husain and Coleman Rotstein (2018). Infections in solid organ transplant recipients. Clin Infect Dis.

[19] Lorenz EC, Cosio FG (2010). The impact of urinary tract infections in renal transplant recipients. Kidney Int.

[20] De Souza RM, Olsburgh J (2008). Urinary tract infection in the renal transplant patient. Nat Clin Pract Nephrol.

[21] Fiorante S, Lopez-Medrano F, Lizasoain M, Lalueza A, Juan RS, Andrés A (2010). Systematic screening and treatment of asymptomatic bacteriuria in renal transplant recipients. Kidney Int.

[22] Pelle G, Vimont S, Levy PP, Hertig A, Ouali N, Chassin C (2007). Acute pyelonephritis represents a risk factor impairing long-term kidney graft function. Am J Transplant.

[23] Kotagiri P, Chembolli D, Ryan J, Hughes PD, Toussaint ND (2017). Urinary tract infections in the first year post–kidney transplantation: potential benefits of treating asymptomatic bacteriuria. Transplant Proc.

[24] Shams SF, Eidgahi ES, Lotfi Z, Khaledi A, Shakeri S, Sheikhi M (2017). Urinary tract infections in kidney transplant 1st year after transplantation. J Res Med Sci.

[25] Bispo A, Fernandes M, Toscano C, Marques T, Machado D, Weigert A (2014). Urinary tract infections in a cohort of kidney transplant recipients. Acta Medica Port.

[26] Alkatheri AM (2013). Urinary tract infections in Saudi renal transplant recipients. J Infect Dis Immun.

[27] Sadeghi M, Daniel V, Naujokat C, Wiesel M, Hergesell O, Opelz G (2005). Strong inflammatory cytokine response in male and strong anti-inflammatory response in female kidney transplant recipients with urinary tract infection. Transpl Int.

[28] Ciszek M, Paczek L, Bartlomiejczyk I, Mucha K (2006). Urine cytokines profile in renal transplant patients with asymptomatic bacteriuria. Transplant Proc.

[29] Becerra BJ, Becerra MB, Safdar N (2015). A nationwide assessment of the burden of urinary tract infection among renal transplant recipients. J Transp Secur.

[30] Kumar A, Agarwal C, Hooda AK, Ojha A, Dhillon M, Hari Kumar KV (2016). Profile of infections in renal transplant recipients from India. J Family Med Prim Care.

[31] Menegueti MG, Pereira MF, Bellissimo-Rodrigues F, Garcia TMP, Saber LTS, Nardim MEP (2015). Study of the risk factors related to acquisition of urinary tract infections in patients submitted to renal transplant. Rev Soc Bras Med Trop.

[32] Elkehili I, Kekli A, Zaak A, Salem E (2010). Urinary tract infection in renal transplant recipients. Arab J Urol.

[33] Ooms L, Ijzermans J, Voor H, Betjes M, Vos M, Terkivatan T (2017). Urinary tract infections after kidney transplantation: a risk factor analysis of 417 patients. Ann Transplant.

[34] Veroux M, Giuffrida G, Corona D, Gagliano M, Scriffignano V, Vizcarra D (2008). Infective complications in renal allograft recipients: epidemiology and outcome. Transplant Proc.

[35] Valera B, Gentil MA, Cabello V, Fijo J, Cordero E, Cisneros JM (2006). Epidemiology of urinary infections in renal transplant recipients. Transplant Proc.

[36] Papasotiriou M, Savvidaki E, Kalliakmani P, Papachristou E, Marangos M, Fokaefs E (2011). Predisposing factors to the development of urinary tract infections in renal transplant recipients and the impact on the long-term graft function. Ren Fail.

[37] Flores-Mireles AL, Walker JN, Caparon M, Hultgren SJ (2015). Urinary tract infections: epidemiology, mechanisms of infection and treatment options. Nature REV Microbiol.

[38] Abbott KC, Swanson SJ, Richter ER, Bohen EM, Agodoa LY, Peters TG (2004). Late urinary tract infection after renal transplantation in the United States. Am J Kidney Dis.

[39] Yuan X, Liu T, Wu D, Wan Q (2018). Epidemiology, susceptibility, and risk factors for acquisition of MDR/XDR gram-negative bacteria among kidney transplant recipients with urinary tract infections. Infect Drug Resist.

[40] Ndemera H, Bhengu B (2017). Factors contributing to kidney allograft loss and associated consequences among post -kidney transplant patients. HSJ.

[41] Bodro M, Sanclemente G, Lipperheide I, Allali M, Marco F, Bosch J (2015). Impact of urinary tract infections on short-term kidney graft outcome. Clin Microbiol Infect.

[42] Magiorakos AP, Srinivasan A, Carey RB, Carmeli Y, Falagas ME, Giske CG (2012). Multidrug-resistant, extensively drug-resistant and pan drug-resistant bacteria: an international expert proposal for interim standard definitions for acquired resistance. Clin Microbiol Infect.

[43] Adamska Z, Karczewski M, Cichanska L (2015). Bacterial infections in renal transplant recipients. Transplant Proc.

[44] Pesce Francesco, Martino Marida, Fiorentino Marco, Rollo Tiziana, Simone Simona, Gallo Pasquale, Stallone Giovanni, Grandaliano Giuseppe, Schena Antonio, Margiotta Marcella, Mininni Donata, Palieri Rita, Lucarelli Giuseppe, Battaglia Michele, Gesualdo Loreto, Castellano Giuseppe (2019). Recurrent urinary tract infections in kidney transplant recipients during the first-year influence long-term graft function: a single-center retrospective cohort study. Journal of Nephrology.

[45] Naik AS, Dharnidharka VR, Schnitzler MA, Brennan DC, Segev DL, Axelrod D (2016). Clinical and economic consequences of first-year urinary tract infections, sepsis and pneumonia in contemporary kidney transplantation practice. Transpl Int.

[46] Yacoub R, Akl NK (2011). Urinary tract infections and asymptomatic bacteriuria in renal transplant recipients. J Glob Infect Dis.

