# Supplementary material for: Bacterial urinary tract infection among adult renal transplant recipients at St. Paul’s hospital millennium medical college, Addis Ababa, Ethiopia
Source: BMC Nephrol. 2019 Jul 31;20:289. doi: 10.1186/s12882-019-1485-9 (PMC6668100; doi:10.1186/s12882-019-1485-9)
Supplement: Supplementary file 1 — Table S1. Significant bacteriuria from urine culture of renal transplant recipients. (DOCX 14 kb) [file 12882_2019_1485_MOESM1_ESM.docx]

Additional file 1: **Table S1** Significant bacteriuria from urine culture of renal transplant recipients.

| **Significant bacteriuria** | **Frequency(n)** | | **Percent (%)** | | |
| --- | --- | --- | --- | --- | --- |
| Yes | 11 | | 14.9 | | |
| No | 63 | | 85.1 | | |
| Total | 74 | | 100 | | |
| **Renal transplant recipients** | **Significant bacteriuria** | |  | | |
|  | **Yes** | **No** | **Total** | **COR(95%CI)** | **P-value** |
| Asymptomatic no (%) | 8(22.2) | 28(77.8) | 36(48.7) | 1.01(0.547-3.829) | 0.847 |
| Symptomatic no (%) | 3(7.9) | 35(92.1) | 38(51.3) |  |  |
| Total no (%) | 11(14.9) | 63(85.1) | 74(100) |  |  |
