# Supplementary material for: Bacterial urinary tract infection among adult renal transplant recipients at St. Paul’s hospital millennium medical college, Addis Ababa, Ethiopia
Source: BMC Nephrol. 2019 Jul 31;20:289. doi: 10.1186/s12882-019-1485-9 (PMC6668100; doi:10.1186/s12882-019-1485-9)
Supplement: Supplementary file 2 — Table S2. Bacterial species isolated from asymptomatic and symptomatic UTI among renal transplant recipients, St Paul’s Hospital Millennium medical college, Addis Ababa, Ethiopia. (DOCX 14 kb) [file 12882_2019_1485_MOESM2_ESM.docx]

Additional file 2: **T****able S2** Bacterial species isolated from asymptomatic and symptomatic UTI among renal transplant recipients, St Paul’s Hospital Millennium medical college, Addis Ababa, Ethiopia.

| **Bacterial isolates** | **Asymptomatic RTX no (%)** | **Symptomatic RTX no (%)** | **Total (%)** |
| --- | --- | --- | --- |
| **Gram-negative** | **5(62.5)** | **0(0.0)** | **5(45.4)** |
| *E.coli* | 2(25) | 0(0.0) | 2(18.18) |
| *Acinetobacter spp.* | 2(25) | 0(0.0) | 2(18.18) |
| *P.mirabilis* | 1(12.5) | 0(0.0) | 1(9.1) |
| **Gram-positive** | **3(37.5)** | **3(99.9)** | **6(54.6)** |
| *Enterococcus spp.* | 1(12.5) | 1(33.3) | 2(18.18) |
| CoNS | 1(12.5) | 1(33.3) | 2(18.18) |
| *S.aureus* | 1(12.5) | 1(33.3) | 2(18.18) |
| **Total** | **8(72.7)** | **3(27.3)** | **11(100)** |

**CoNS**=Coagulase Negative *Staphylococci*
