# Supplementary material for: Bacterial urinary tract infection among adult renal transplant recipients at St. Paul’s hospital millennium medical college, Addis Ababa, Ethiopia
Source: BMC Nephrol. 2019 Jul 31;20:289. doi: 10.1186/s12882-019-1485-9 (PMC6668100; doi:10.1186/s12882-019-1485-9)
Supplement: Supplementary file 3 — Table S3. Antibacterial susceptibility patterns of Gram-positive bacterial isolates. (DOCX 17 kb) [file 12882_2019_1485_MOESM3_ESM.docx]

Additional file 3: **Table S3** Antibacterial susceptibility patterns of Gram-positive bacterial isolates.

| **Urine Culture out put** | | | **Antibacterial agents tested** | | | | | | | | | | |
| --- | --- | --- | --- | --- | --- | --- | --- | --- | --- | --- | --- | --- | --- |
|  |  |  | **VA** | **CTR** | **CHL** | **NIT** | **AMC** | **PEN** | **TET** | **SXT** | **ERY** | **CLN** | **CN** |
| Bacterial isolates | Total | Pattern | no(%) | no(%) | no(%) | no(%) | no (%) | no(%) | no(%) | no(%) | no (%) | no (%) | no (%) |
| *S. aureus*  (n=2) | 2 | R  I  S | 0(0)  2(100  0(0) | 2(100)  0(0)  0(0) | 2(100)  0(0)  0(0) | 2(100) 0(0)  0(0) | 1(50)  0(0)  1(50) | 2(100  0(0)  0(0) | 1(50)  0(0) 1(50 | 1(50) 0(0) 1(50) | 0(0)  0(0)  2(100 | 0(0)  0(0)  2(100) | 0(0)  0(0)  2(100) |
| CoNS  (n=2) | 2 | R  I  S | 1(50)  0(0)  1(50) | 1(50)  1(50)  0(0) | 2(100)  0(0) 0(0) | 2(100)  0(0)  0(0) | 2(100  0(0)  0(0) | 0(0)  1(50)  1(50) | 2(100  0(0)  0(0) | 0(0)  1(50)  1(50) | 0(0)  2(100 0(0) | 0(0)  0(0)  2(100) | 2(100)  0(0)  0(0) |
| *Enterococcus spp.*(n=2) | 2 | R  I  S | 0(0)  2(100  0(0) | 2(100)  0(0)  0(0) | 0(0)  1(50)  1(50) | 0(0)  0(0)  2(100) | 2(100  0(0)  0(0) | 1(50)  0(0)  1(50) | 1(50)  0(0)  1(50) | 2(100  0(0)  0(0) | 2(100  0(0)  0(0) | 2(100  0(0)  0(0) | 2(100)  0(0)  0(0) |
| Total  (n=6) | 6 | R  I  S | 1(17)  4(67)  1(17) | 5(83)  1(17)  0(0) | 4(67)  1(17)  1(17) | 4(67)  0(0)  2(33) | 5(83)  0(0)  1(17) | 3(50)  1(17)  2(33) | 4(67)  0(0)  2(33) | 3(50)  1(17)  2(33) | 2(33)  2(33)  2(33) | 2(33)  0(0)  4(67) | 4(67)  0(0)  2(33) |

**Abbreviations:** **R** = Resistant S = Sensitive **I** = Intermediate, **AMC**= Amoxicillin Clavulanate acid, **CTR**=Ceftriaxone, **CHL**=Chloramphenicol, **CLN**=Clindamycin, **ERY**=Erythromycin, **CN**=Gentamicin, **NIT**= Nitrofurantoin, **TET**=Tetracycline, **SXT**=Trimethoprim-Sulfamethoxazole, **VA**= Vancomycin, **PEN** =Penicillin
