# Supplementary material for: Bacterial urinary tract infection among adult renal transplant recipients at St. Paul’s hospital millennium medical college, Addis Ababa, Ethiopia
Source: BMC Nephrol. 2019 Jul 31;20:289. doi: 10.1186/s12882-019-1485-9 (PMC6668100; doi:10.1186/s12882-019-1485-9)
Supplement: Supplementary file 4 — Table S4. Antibacterial susceptibility patterns of Gram-negative bacteria isolates. (DOCX 17 kb) [file 12882_2019_1485_MOESM4_ESM.docx]

Additional file 4: **Table S4** Antibacterial susceptibility patterns of Gram-negative bacteria isolates.

| **Urine Culture out put** | | | **Antibacterial agents tested** | | | | | | | | | | | |
| --- | --- | --- | --- | --- | --- | --- | --- | --- | --- | --- | --- | --- | --- | --- |
|  |  |  | **CIP** | **TET** | **CN** | **AMC** | **CHL** | **NIT** | **SXT** | **CPM** | **CTR** | **MEM** | **DC** |  |
| Bacterial isolates | Total | Pattern | no (%) | no (% | no(%) | no(% | no(% | no(%) | no(% | no(% | no(% | no(%) | no(% |  |
| *E.coli* (n=2) | 2 | R  I  S | 2(100 0(0)  0(0) | 1(50  0(0) 1(50) | 0(0)  0(0)  2(100 | 1(50)  1(50)  0(0) | 2(100  0(0)  0(0) | 2(100  0(0)  0(0) | 2(100  0(0)  0(0) | 2(100 0(0)  0(0) | 2(100  0(0)  0(0) | 0(0) 2(100) 0(0) | 1(50)  0(0)  1(50) |  |
| *Acinetobacter spp*.(n=2 | 2 | R  I  S | 2(100  0(0 0(0) | 0(0)  0(0) 2(100 | 0(0)  0(0)  2(100 | 2(100  0(0 0(0 | 2(1000(0) 0(0) | 2(100  0(0)  0(0) | 2(100  0(00)  1(50) | 1(50)  0(0) 1(50) | 2(100  0(0)  0(0) | 1(50)  1(50)  0(0) | 0(0)  0(0)  2(100 |  |
| *P. mirabilis* (n=1) | 1 | R  I  S | 0(0) 0(0)  1(100 | 1(100 0(0)  0(0) | 0(0)  0(0) 1(10 | 0(0)  0(0  (100 | 0(0)  1(00)  0(0) | 1(100  0(0)  0(0) | 0(0)  1(100 0(0) | 0(0)  0(0  1(100 | 0(0)  0(0)  1(100 | 0(0)  0(0)  1(100 | 1(100  0(0) 0(0) |  |
| Total (n=5) | 5 | R  I  S | 4(80)  0(0)  1(20) | 2(40)  0(0)  3(60) | 0(0)  0(0)  5(100 | 3(60)  1(20)  1(20) | 4(80) 1(20 )  0(0) | 5(100  0(0)  0(0) | 4(80)  1(20)  0(0) | 3(60)  0(0)  2(40) | 4(80) 0(0) 1(20) | 1(20) 3(60) 1(20) | 2(40)  0(0)  3(60) |  |

**Abbreviations**: **R** = Resistant, **S** = Sensitive, **I** = Intermediate, **AMC**= Amoxicillin-Clavulanate acid, **CFM**=Cefepime,**CTR**=Ceftriaxone,**CHL**=Chloramphenicol, **CIP**= Ciprofloxacin**,** **CN**=Gentamicin , **MER**=Meropenem, **NIT**=Nitrofurantoin, **TET**=Tetracycline, **SXT**=Trimethoprim-Sulfamethoxazole, **DC**=Doxycycline.
